# Treatment adherence among sputum smear-positive pulmonary tuberculosis patients in Xinjiang, China: a prospective study

**DOI:** 10.1039/c7ra11820a

**Published:** 2018-02-28

**Authors:** Xinji Gong, Yuehua Li, Jing Wang, Gang Wu, Ayinuer Mohemaiti, Qimanguli Wushouer, Lidan Yao, Jianghua Lv, Rongrong Li, Pengfei Li, Buqing Wang

**Affiliations:** Department of Respiratory Diseases, The First Affiliated Hospital of Xinjiang Medical University Urumqi China; Wuhan City Pulmonary Hospital (Wuhan Tuberculosis Control Institute) Wu Han China; Department of Geriatrics, The First Affiliated Hospital of Xinjiang Medical University Urumqi China tlfwj@163.com; Xinjiang Uygur Autonomous Region Center for Disease Control and Prevention Urumqi China; Department of Nutrition, The First Affiliated Hospital of Xinjiang Medical University Urumqi China

## Abstract

Background: Despite great effort to control tuberculosis (TB), low treatment adherence threatens the success of drug therapy, increases the risk of TB transmission, and leads to the development of drug resistance. The present study assessed anti-TB treatment adherence in sputum smear-positive TB patients and examined the risk factors for poor patient adherence to identify targets for intervention. Methods: We monitored and followed up TB patients who were diagnosed between July 2014 and June 2015 in Xinjiang, China. A total of 8289 sputum smear-positive TB patients were included in this study. All patients registered their information during the first hospital visit or with the Centers for Disease Control, had regular follow-up visits, and accepted the anti-TB treatment. Insufficient re-examination adherence was defined as undergoing fewer than the recommended three sputum smear examinations during the treatment course. Results: Among 8289 patients, 3827 men (84.4% of male patients) and 3220 women (85.7% of female patients) had good adherence during treatment follow-up. 1242 patients (15.0%) did not complete regular follow-up. 332 (4.0%) patients lost contact. An adjusted logistic regression model showed that ethnicity, household address, treatment classification, patient source, and the actual management were significantly associated with non-adherence. Conclusion: The Xinjiang TB epidemic situation remains grim. Smear-positive patients had a higher proportion of non-adherence, which increased treatment difficulties and the risk of death from TB. Relevant medical departments should strengthen their supervision and interventions during the TB treatment process to improve patient adherence to anti-TB treatment.

## Abstract 2

Background: Despite great effort to control tuberculosis (TB), low treatment adherence threatens the success of drug therapy, increases the risk of TB transmission, and leads to the development of drug resistance.

## Graphical abstract

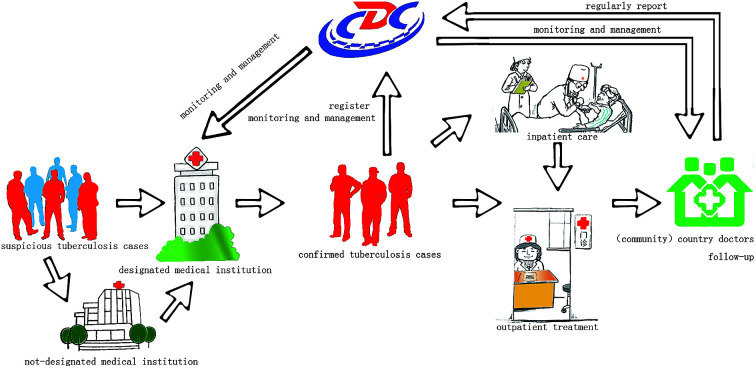

## Introduction

Tuberculosis (TB) causes significant morbidity and mortality and remains a major global health problem.^1^ According to the 2015 World Health Organization (WHO) Global TB Control Report, 10.4 million new cases of TB were diagnosed in 2015. There were an estimated 1.4 million TB deaths in 2015, and an additional 0.4 million deaths resulted from TB disease among people living with HIV. However, globally, there were 4.3 million more actual cases of disease than reported.^2^ Currently, approximately one-third of the world's population is infected with TB bacteria.^3^ Therefore, TB can have far-reaching economic and social consequences among infected people and their household members, particularly in developing countries.^4^

China accounts for 15% of the global burden of TB and has the third highest TB incidence rate in the world behind India and Indonesia.^2^ Data from the Fifth National TB Epidemiological Survey in 2010 showed that China's active TB prevalence was 459/100 000, with 66/1 000 000 representing sputum smear-positive TB.^5^ The Xinjiang Uyghur Autonomous Region (Xinjiang) is located in northwestern China, with international borders with Russia, Mongolia, Kazakhstan, Kyrgyzstan, Tajikistan, Afghanistan, Pakistan, and India, with a population of 20 million people and 13 ethnic minorities.^6^ Xinjiang is one of the provinces in China with a high burden of TB.^7^ Xinjiang is one of the provinces with high burden of TB in China. Its economy is relatively backward, its public health infrastructure is weak and its medical resources are scarce. Many problems exist in the process of diagnosis, treatment and management of TB, which leads to the high number of patients and their constant transmission has become the thorniest challenge in tuberculosis control in China.

Within the international TB control strategy, direct observation of treatment (DOT) was introduced to improve treatment outcomes. A standard anti-TB treatment requires patients to regularly take a complex combination of drugs for at least 6 months for new patients and 8 or 9 months for those undergoing repeat retreatment. Such long-term strict regimens are a challenge for TB patients who may not adhere to their prescribed treatment due to treatment interruptions or drop-out.^8^ Adherence to treatment is crucial to achieving a successful treatment outcome.^9^ Poor adherence to treatment may prolong infectiousness and increase the risk of drug resistance, relapse, death, and onward transmission.^10^ Rocha *et al.* showed that treatment non-adherence was significantly associated with unfavorable outcomes (death or failure to cure TB).^11^ Other studies on adherence have shown improved TB cure rates.^12,13^ In recent years, many cities in China have performed studies of TB treatment adherence, and the results indicated low treatment adherence among smear-positive TB patients.^14–16^

We conducted surveillance and follow-up of TB treatment in major cities in Xinjiang to evaluate patient compliance with anti-TB treatment and to determine the risk factors of non-adherence in smear-positive TB patients. This study provides a theoretical basis for the next step in developing measures to improve patient compliance in accordance with the TB situation in Xinjiang.

## Methods

### Pulmonary tuberculosis diagnosis

The diagnosis of pulmonary TB patients in Xinjiang was based on the People's Republic of Health Industry standard diagnostic criteria for TB (WS288-2008). Patients were considered to have smear-positive pulmonary TB when they met one of the following 3 criteria: 2 sputum specimens directly showing smear-positive acid-fast bacilli; 1 sputum specimen directly showing acid-fast bacilli with a positive microscopic examination and pulmonary imaging in line with the performance of active pulmonary TB imaging; or 1 sputum specimen directly showing smear-positive acid-fast bacilli and 1 sputum specimen that was *Mycobacterium tuberculosis* culture-positive.

### Pulmonary tuberculosis treatment

The treatment regimen for new cases of TB consisted of a two-month intensive phase with a daily dose of fixed-dose combination tablets containing rifampicin (R), isoniazid (H), pyrazinamide (Z), and ethambutol (E), followed by a four-month continuation phase of daily HR. New patients underwent three re-examinations for sputum smear microscopy to monitor treatment response after the second, fifth, and sixth months of treatment. The repeat treatment patients underwent a three-month intensive phase including the four drugs above along with a six-month continuation phase of daily HRE. At the end of the course of 2, 5, or 8 months, the patients returned for re-examinations. Smear-positive patients are expected to convert to negative status after two months of intensive treatment. During the 2 month treatment period, if the patient's sputum smears were still positive, they were prescribed an additional sputum smear at the end of the third month and an additional month of the intensive phase treatment.

### TB management

The tuberculosis management in Xinjiang uses the whole process of supervision and management, since the 2012 implementation of the “Trinity” new TB prevention and control of new models, a prevention and treatment service system of designated medical institutions, primary health care institutions and the CDC, with clear division and coordination. Among these facilities, the designated medical institutions are responsible for the diagnosis, treatment, and registration of TB patients. Non-designated medical institutions are responsible for the initial screening, epidemic reporting, and referral of TB patients. Primary health care institutions are responsible for patient referrals and helping track and follow-up with patients. The CDC is responsible for planning, coordinating, monitoring, and controlling the response to the epidemic. In addition, the CDC is also responsible for prevention and technical guidance, publicity and education, performance evaluation, and other measures.

### Study design

The data were obtained from the quarterly and annual reports of the Xinjiang Tuberculosis Management Information System. All diagnosed cases of active TB, new TB pleurisy, and extra-pulmonary TB were registered by the designated medical institutions and county CDC, which recorded the basic information and treatment outcomes with regular follow-up and supervision. Simultaneously, the primary health care institutions monitored and reviewed the patients' medications and other health measures.

After combining the patients' case records with the treatment records in the report charts, we established statistical tables of treatment adherence for smear-positive TB patients in Xinjiang. We identified the population as smear-positive TB patients from July 2014 to June 2015 and collected basic information about the patients and their re-examinations at the end of second, fifth, and sixth (eighth) months and the treatment outcomes. To protect patient privacy, all information omitted the patient's name and ID number. We defined good adherence as the completion of three sputum smear re-examinations and non-adherence as failure to complete three sputum smear re-examinations. The treatment outcomes included cure, treatment completion, tuberculosis death, failure, and drop-out.^17^ At the same time, we defined a clinical cure as disease-free status and treatment completion. We registered basic patient information including age, gender, ethnicity, occupation, household address, treatment classification, patient source, diagnosis patterns, actual management methods, *etc.*

By integrating the above data, we described the basic information for the smear-positive patients in Xinjiang from July 2014 to June 2015, explored the correlation between compliance and the cure rate and survival rate of smear-positive TB patients and discussed the factors that influence treatment compliance.

### Statistical analysis

The data were double-entered into Excel 2010 and analyzed using SPSS 18.0 for Windows by two procedures. Chi-squared tests were used to identify associations between patient adherence and the cure rate or death rate. Univariate logistic regression was used to analyze the relationship between each variable and adherence to assess independent risk factors for non-adherence. Variables with a *P*-value <0.05 in the univariate analysis were included in the multivariable logistic regression models. The magnitude of association was measured by the odds ratio (OR) with a confidence interval (CI). The level of significance was set at *P* < 0.05.

### Ethical considerations

All experiments were performed in compliance with medical ethics, and approved by the ethics committee at Xinjiang Medical University. Informed consents were obtained from human participants of this study.

## Results

This study included 8289 patients with smear-positive cases of TB in Xinjiang from July 2014 to June 2015. The age of the study patients ranged from 5 to 102 years with an average age of 48.9 years. The study included 54.7% males and 45.3% females.

Regarding ethnicity, 14.4% of the patients were Han and 68.7% were Uighur. The local population accounted for 88.3%, and the floating population accounted for 11.7%. The study population was composed mainly of workers, farmers, and those involved in animal husbandry and fishery (78.5%). Newly treated patients represented 74.8% of the sample, and those undergoing retreatment accounted for 25.2%. Type III TB patients accounted for 99.1% of the study population; 1.4% of the patients were diagnosed by health examination, whereas 28.7% and 38.7% were diagnosed by illness or referral. The vast majority of patients (87.4%) were managed in full supervision mode (Table 1).

**Table 1:** Baseline characteristics of 8289 patients with smear-positive tuberculosis in Xinjiang

Basic information	*N* (%)
*Gender*	
Male	4532 (54.7)
Female	3757 (45.3)
*Age*	
∼15	92 (1.1)
∼30	1924 (23.2)
∼45	1607 (19.4)
∼60	1740 (21.0)
>60	2926 (35.3)
*Ethnicity*	
Han	1191 (14.4)
Hui	302 (3.6)
Uyghur	5693 (68.7)
Kazak	862 (10.4)
Other	241 (2.9)
*Permanent residence address*	
Non-mobile population	7323 (88.3)
Floating population	966 (11.7)
*Professional*	
Cadres	147 (1.8)
Professional and technical personnel	113 (1.4)
Food and beverage business	50 (0.6)
Industry, agriculture, animal husbandry and fishery	6510 (78.5)
Former employment or unemployment	1062 (12.8)
Other	407 (4.9)
*Treatment classification*	
Untreated	6204 (74.8)
Retreated	2085 (25.2)
*Diagnostic classification*	
Type I	1 (0.0)
Type II + III	64 (0.8)
Type III	8216 (99.1)
Type IV + III	7 (0.1)
Type V + III	1 (0.0)
*Patient sources*	
Physical examination	116 (1.4)
Visited doctor with symptoms	2382 (28.7)
Because of the disease treatment is recommended	1124 (13.6)
Referral	3204 (38.7)
Tracking	1402 (16.9)
Contacts to check	20 (0.2)
Other	41 (0.5)
*Actual management*	
Entire supervision	7245 (87.4)
Entire management	605 (7.3)
Phase strengthening supervision	162 (2.0)
Self-administration	3 (0.0)
Other	274 (3.3)

Treatment outcomes varied during the study. Cures were attained in 7039 (84.9%) patients. The treatment course was completed by 302 (3.6%) patients. Seven (0.0%) patients developed disease resistant to a single drug, whereas 23 (0.3%) patients were transferred to multi-drug resistant treatment. Ninety-eight (1.2%) patients experienced adverse effects, and 199 (2.4%) patients had treatment failure. Ninety-five (1.1%) patients died of TB, and 332 (4.0%) lost contact during follow-up. One (0.0%) patient was incorrectly diagnosed, and 193 (2.3%) died for other reasons not related to TB. We concluded that the number of clinically cured patients was 7341 (88.6%), excluding those who lost contact, wrongly diagnosed, or died of other diseases (including cardiovascular and cerebrovascular diseases, tumors, *etc.*). Patient compliance and attainment of a clinical cure were positively correlated (*χ*^2^ = 0.536, *P* < 0.001). The difference was statistically significant. The association between the compliance of the population and the outcome of TB death was related (*χ*^2^ = 0.326, *P* < 0.001). The difference was statistically significant (Tables 2 and 3).

**Table 2:** Relationship between compliance and cure rate of smear-positive tuberculosis patients^a^

Compliance	Cure (%)	Non-cure (%)	Total	*χ* ^2^	*P*
Compliance	6877 (98.7)	93 (1.3)	6970		
Non-adherence	464 (58.5)	329 (41.5)	793	*χ* ^2^ = 0.536	*P* < 0.0001
Total	7341	422	7763		

a Note 1: from July 2014 to June 2015, there were 8289 patients in the Xinjiang cohort. Of these, 332 were unsuccessful due to loss, and 193 were died of other diseases. Note 2: clinical cure (cure, complete course of treatment), non-cure (single-drug resistant, multidrug-resistant treatment, adverse reaction, failure, tuberculosis death).

**Table 3:** Relationship between adherence and survival rate for smear-positive tuberculosis patients^a^

Compliance	Death (%)	Non-death (%)	Total	*χ* ^2^	*P*
Compliance	1 (0.0)	6969 (100.0)	6970		
Non-adherence	94 (11.9)	699 (88.1)	793	*χ* ^2^ = 0.326	*P* < 0.0001
Total	95	7668	7763		

a Note 1: from July 2014 to June 2015, there were 8289 patients in the Xinjiang cohort. Of these, 332 were unsuccessful due to loss, 193 were died of other diseases, and 1 was incorrectly diagnosed. Note 2: death (tuberculosis death), non-death (cure, complete course of treatment, single-drug resistance, MDR-resistant, adverse reactions, failure).

We observed 1242 (15.0%) patients who had poor adherence to treatment and failed to complete three sputum smear visits. Treatment adherence was compared among different subgroups using a univariate logistic regression analysis. In this study, we found that those patients with poor adherence were elderly (*P* < 0.01), those from a floating population (*P* < 0.05), manual laborers (*P* < 0.01), those who were derived from referral and tracking (*P* < 0.01), or those who were self-medicated and supervised during the intensive phase. A multivariate logistic regression analysis controlled the effect of confounding factors and analyzed the associated risk factors for non-compliance. Univariate variables with *P* values less than 0.05 were included in the multivariate logistic regression. The results showed that ethnicity, household address, treatment classification, patient source, and actual management were correlated with non-compliance (Table 4).

**Table 4:** Analysis of influential factors of the treatment compliance of 8289 smear-positive tuberculosis patients in Xinjiang

Basic information	*N*	Non-adherence *n* (%)	Univariate analysis			Multivariate analysis		
			OR	95% CI	*P*	OR	95% CI	*P*
*Gender*								
Male	4532	705 (15.6)	1		0.109	1		
Female	3757	537 (15.0)	0.905	0.802–1.022		0.888	0.777–1.014	0.080
*Age*								
∼15	92	7 (7.6)	1		**0.000**	1		
∼30	1924	200 (10.4)	1.409	0.643–3.087		0.890	0.383–2.071	0.787
∼45	1607	177 (11.0)	1.503	0.685–3.300		0.848	0.360–1.998	0.705
∼60	1740	254 (14.6)	2.076	0.949–4.537		1.083	0.461–2.543	0.854
>60	2926	604 (20.6)	3.159	1.454–6.862		1.628	0.697–3.797	0.261
*Ethnics*								
Han	1191	88 (7.4)	1		**0.000**	1		
Hui	302	16 (0.2)	0.701	0.405–1.213		0.777	0.439–1.378	0.388
Uyghur	5693	1032 (12.5)	2.775	2.211–3.484		3.010	2.330–3.890	**0.000**
Kazak	862	76 (0.9)	1.212	0.880–1.669		1.677	1.180–2.384	**0.004**
Other	241	30 (0.4)	1.782	1.148–2.766		1.926	1.188–3.122	**0.008**
*Household address*								
Non-mobile population	7323	1072 (14.6)	1		**0.016**	1		
Floating population	966	170 (17.6)	1.245	1.042–1.488		1.329	1.071–1.649	**0.010**
*Professional*								
Cadres	147	15 (10.2)	1		**0.000**	1		
Professional and technical personnel	113	11 (9.7)	0.949	0.418–2.154		0.835	0.346–2.014	0.689
Food and beverage business	50	6 (12.0)	1.200	0.439–3.283		1.290	0.419–3.973	0.657
Industry, agriculture, animal husbandry and fishery	6510	1033 (15.9)	1.660	0.969–2.843		1.004	0.564–1.787	0.990
Former and unemployment	1062	148 (13.9)	1.425	0.813–2.499		1.195	0.656–2.176	0.561
Other	407	29 (7.1)	0.675	0.351–1.299		0.722	0.353–1.475	0.371
*Treatment classification*								
Untreated	6204	756 (12.2)	1		**0.000**	1		
Retreated	2085	486 (23.3)	2.190	1.929–2.487		1.653	1.433–1.907	**0.000**
Diagnostic classification								
Type III	8216	1233 (15.0)	1		0.524			
Other	73	9 (0.1)	0.796	0.395–1.604				
*Patients sources*								
Physical examination	116	18 (15.5)	1		**0.000**	1		
Visited doctor with symptoms	2382	285 (12.0)	0.740	0.441–1.242		4.117	2.106–8.047	**0.000**
Because of the disease treatment is recommended	1124	139 (12.4)	0.768	0.451–1.309		3.013	1.526–5.949	**0.001**
Referral	3204	528 (16.5)	1.074	0.644–1.791		6.326	3.247–12.325	**0.000**
Tracking	1402	260 (18.5)	1.240	0.737–2.086		6.513	3.307–12.830	**0.000**
Contacts to check	20	2 (10.0)	0.605	0.129–2.836		3.416	0.552–21.133	0.186
Other	41	10 (24.4)	1.756	0.734–4.201		7.257	2.587–20.354	**0.000**
*Actual management*								
Entire supervision	7245	929 (12.8)	1		**0.000**	1		
Entire management	605	40 (6.6)	0.481	0.347–0.668		0.409	0.293–0.572	**0.000**
Phase strengthening supervision	162	75 (46.3)	5.861	4.271–8.042		6.214	4.453–8.673	**0.000**
Self-administration	3	1 (33.3)	3.399	0.308–37.526		3.696	0.330–41.377	0.289
Other	274	197 (71.9)	17.394	13.248–22.837		19.967	14.551–27.397	**0.000**

## Discussion

TB threatens public health,^18^ and its prevalence hinders economic and social development. China is one of the 20 countries with highest proportion of TB cases worldwide as the “Stop TB Strategy” of the WHO,^19^ and Xinjiang has a serious TB epidemic situation. Effective treatment is essential to controlling the development and spread of TB; however, because anti-TB treatment requires long-term drug therapy, patients are prone to loss of compliance.^20,21^ Follow-up visits during treatment are important to monitor the response to the therapy and manage adverse events, which facilitates the supervision of patient treatment and prevention of lost cases.^22^

In our study, approximately 15.0% of the patients were poorly adherent. This proportion was significantly higher than that of the Jiangsu (11.4%), Anhui (9.5%), and Ningxia (6.7%) patients.^16^ We also found that compliance was related to the level of TB cure rate and mortality. The cure rate of the compliance group (98.7%) was significantly higher than that of the non-compliance group (58.5%), and the mortality in the compliance group (0.0%) was significantly lower than in the non-compliance group (11.9%). Thus, it is necessary to improve the cure rate of the smear-positive population and reduce mortality by improving patient treatment compliance. However, recent Chinese studies have shown that most TB patients are unaware of the importance of return visits.^23^

In the study, we found that gender and age were not associated with patient adherence to treatment, which was consistent with the results of previous studies.^24^ However, some studies have shown that male patients may be more likely to not comply with treatment because of their unhealthy lifestyles and physical over-confidence.^25^ The proportion of smear-positive TB patients in Xinjiang is high (85.6%), and the proportion of non-compliance among ethnic minorities is high. The Uighur patients accounted for 83.1% of the total number of non-compliant individuals. On the one hand, the high incidence of TB in Xinjiang ethnic minorities may be associated with the presence of TB susceptibility genes.^26,27^ On the other hand, most ethnic minorities lack TB-related knowledge and have not yet recognized the importance of long-term anti-TB treatment. Once the symptoms ease, these patients will be without medication. Some patients live in remote areas or in the country where treatment and inspection are not very convenient, resulting in many TB patients who cannot adhere to treatment and referral. We found that migrants were at risk for noncompliance, and 966 (11.7%) of the study population were migrants, with 17.6% being classified as non-adherent. Most of the floating population also lacks a fixed place, cannot adhere to timely referrals, are prone to treatment delays, and are not easy to manage and monitor. This study showed that adherence was significantly lower in newly treated patients than in retreated patients. The reason for this may be that re-treated patients can encounter drug resistance. During the retreatment process, these patients may lack confidence in treatment, causing the re-treatment course to be longer. In this case, the symptoms may not improve, which can also lead to poor compliance. This study found that the patient source, along with patient referral and tracking, were significantly associated with compliance, possibly due to the fact that healthy examined people are more concerned about their own physical condition, which results in greater treatment compliance. The referral and tracking of patients as recommended by the CDC intervention may result in lower patient initiative for treatment. This study also found that intensive phase supervision is a risk factor for patient compliance. Although some patients in the intensive phase have very good compliance under supervision, if the publicity and supervision are insufficient during the continuing period, intermittent treatment and irregular follow-up may result, which will affect the treatment. Research has also shown that during the entire process of supervision and management, patients have good treatment compliance.

In recent years, there has been a high incidence of new cases of TB worldwide because of the increased population, the floating population, the increased incidence of drug-resistant TB, latent infection, and the AIDS epidemic.^28,29^ In 2015, the WHO proposed a moratorium on TB that aimed to reduce the incidence of TB by 90% by 2035.^30^ At present, there is no early diagnostic procedure for TB, leading to delayed treatment in most TB patients.^31^ The number of physical workers in the study accounted for 78.5% of the total number of smear-positive patients, and most of these individuals lacked basic knowledge of TB. It is easy to delay healthcare until symptoms appear, which results in delayed treatment.

Some studies have shown that family care and support played an important role during TB treatment.^32^ However, the remaining TB patients represent a serious psychological burden and stress. We believe that grass-roots medical institutions should strengthen the basic knowledge of TB-related advocacy, improve patient awareness of TB, and provide some psychological support for those patients undergoing repeat treatment. Family members should be encouraged to care for one another and supervise patient medication and regular referral. To reduce the financial burden of TB patients, the state provides TB patients with free anti-TB drugs and necessary inspection items, and although this free policy covers all of the typical TB patients, it does not include patients with adverse reactions to treatment or drug-resistant patients who require second-line anti-TB drugs. However, the treatment enthusiasm is not high for some farmers and herdsmen in remote areas because of the distance and the extra cost.^16^ In this regard, the relevant government departments should expand their focus on supporting this part of the population.

## Limitations

There are still some limitations of this study. Compliance for TB treatment includes medication adherence and compliance, and this study discussed only the factors that influence compliance. The basic patient information did not cover economic and cultural factors.

## Conclusion

The Xinjiang TB epidemic situation remains grim. Smear-positive patients had a higher proportion of non-adherence. Ethnicity, residence address, classification, treatment regimen, and the actual source of management were associated with non-compliance. Non-compliance increased the difficulty of treatment and risk of death. The relevant medical departments should strengthen the supervision and intervention of the TB treatment process, strengthen TB-related basic knowledge propaganda, raise awareness of TB patients, and give financial and policy support to farmers and herdsmen in remote areas to improve anti-TB treatment adherence.

## Conflicts of interest

All authors do not have a conflict of interest.

## Supplementary Material
